# Coronary Revascularization and Long-Term Survivorship in Chronic Coronary Syndrome

**DOI:** 10.3390/jcm10040610

**Published:** 2021-02-05

**Authors:** Ana Gabaldon-Perez, Victor Marcos-Garces, Jose Gavara, Cesar Rios-Navarro, Gema Miñana, Antoni Bayes-Genis, Oliver Husser, Juan Sanchis, Julio Nunez, Francisco Javier Chorro, Vicente Bodi

**Affiliations:** 1 Department of Cardiology, Hospital Clínico Universitario de Valencia, 46010 Valencia, Spain; anagabaldonperez@gmail.com (A.G.-P.); vic_mg_cs@hotmail.com (V.M.-G.); gemineta@gmail.com (G.M.); sanchis_juafor@gva.es (J.S.); yulnunez@gmail.com (J.N.); francisco.j.chorro@uv.es (F.J.C.); 2 INCLIVA Health Research Institute, 46010 Valencia, Spain; jose_4_6_90@hotmail.com (J.G.); cesar_rios1@hotmail.com (C.R.-N.); 3 Center for Biomaterials and Tissue Engineering, Universitat Politècnica de València, 46022 Valencia, Spain; 4 Centro de Investigación Biomédica en Red—Cardiovascular (CIBER-CV), 28029 Madrid, Spain; abayesgenis@gmail.com; 5 Department of Medicine, Faculty of Medicine and Odontology, Universidad de Valencia, 46010 Valencia, Spain; 6 Cardiology Department and Heart Failure Unit, Hospital Universitari Germans Trias i Pujol, 08916 Badalona, Spain; 7 Department of Medicine, Universitat Autonoma de Barcelona, 08193 Barcelona, Spain; 8 Department of Cardiology, St-Johannes Hospital, 44137 Dortmund, Germany; oliver.husser@gmail.com

**Keywords:** ischemic heart disease, chronic coronary syndrome, coronary artery disease, myocardial revascularization, ischemia, stress cardiac magnetic resonance

## Abstract

Ischemic heart disease (IHD) persists as the leading cause of death in the Western world. In recent decades, great headway has been made in reducing mortality due to IHD, based around secondary prevention. The advent of coronary revascularization techniques, first coronary artery bypass grafting (CABG) surgery in the 1960s and then percutaneous coronary intervention (PCI) in the 1970s, has represented one of the major breakthroughs in medicine during the last century. The benefit provided by these techniques, especially PCI, has been crucial in lowering mortality rates in acute coronary syndrome (ACS). However, in the setting where IHD is most prevalent, namely chronic coronary syndrome (CCS), the increase in life expectancy provided by coronary revascularization is controversial. Over more than 40 years, several clinical trials have been carried out comparing optimal medical treatment (OMT) alone with a strategy of routine coronary revascularization on top of OMT. Beyond a certain degree of symptomatic improvement and lower incidence of minor events, routine invasive management has not demonstrated a convincing effect in terms of reducing mortality in CCS. Based on the accumulated evidence more than half a century after the first revascularization procedures were used, invasive management should be considered in those patients with uncontrolled symptoms despite OMT or high-risk features related to left ventricular function, coronary anatomy, or functional assessment, taking into account the patient expectations and preferences.

## 1. Chronic Coronary Syndrome: Definition and Perspective

Ischemic heart disease (IHD) refers to an inadequate supply of blood to the myocardium, in most cases owing to arteriosclerotic plaques in the coronary arteries. Most patients can be given the diagnosis of chronic coronary syndrome (CCS), also referred to as stable IHD. With many exceptions, clinical presentation typically consists of a classic history of angina pectoris in the presence of cardiovascular risk factors, occurring predictably and reproducibly at a certain level of exertion, and relieved with rest or nitroglycerin [1].

During the last few decades, the effect of coronary revascularization on long-term survivorship in patients with CCS has been a matter of constant debate [2]. A *déjà vu* of studies has led to a degree of skepticism, and against this background a series of factors need further consideration. The effect of treatment in patients under-represented in trials such as those with no or mild ischemia, or conversely with very severe ischemia, must be interpreted with caution. Data available from large registries, along with the clinical experience accumulated over the decades, need to be examined in detail. Moreover, the balance between lack of overall reduction of death or myocardial infarction (MI) and benefits in terms of minor events or patient-centered outcomes (such as amelioration of symptoms or a reduced need for unplanned procedures) must be weighed up individually during decision-making on treatments that may involve risk with short- and long-term implications.

In the 2020s, in line with the current paradigm in health systems to deliver the most verifiable outcomes, all-cause death has been consolidated as the most robust and unarguable endpoint when evaluating the effect of potentially risky therapies. This manuscript provides a historical perspective of knowledge accumulated on the effect of coronary revascularization on the survivorship of CCS patients over the half century since the first procedures were performed. Based on the available evidence, we then outline our standpoint on the criteria for appropriate use of coronary revascularization in this scenario.

## 2. Surgical Revascularization: “*The Great Hope*”

Coronary surgery developed gradually throughout the 20th century, after taking its earliest steps in 1910 when Alexis Carrel made the first experimental coronary revascularization attempt using the carotid artery in dogs [3]. Arthur Vineberg refined the concept in 1946 by performing a left internal thoracic artery (LITA) implant directly into the front wall of the left ventricle, known as the Vineberg procedure [4]. Later on, the first documented successful coronary artery bypass grafting (CABG) surgery was performed by Robert Goetz in 1960 employing tantalum rings [5]. Shortly afterward, the first direct hand-sewn coronary anastomosis was performed by David Sabiston in 1962. He anastomosed a saphenous vein graft to the right coronary artery (RCA) in a patient who died three days after the intervention [6]. The first successful hand-sewn coronary anastomosis is historically attributed to Vasilii Kolessov two years later [7]. In 1968, George Green performed the first LITA to the left anterior descending (LAD) anastomosis, which has become the gold standard for the CABG surgery [8]. All these contributions ushered in the beginning of evidence-based CABG surgery. Nevertheless, the author who laid the foundations for its future benefits was Argentinian surgeon René Favaloro, who performed his first bypass surgery in 1967 [9] and actively worked in this field throughout his professional career.

During the 1970s, the number of bypass grafting procedures was increasing rapidly, and use of CABG surgery was first proposed for patients with CCS. Several randomized clinical trials were designed comparing an initial medical therapy strategy with CABG surgery [10,11,12,13,14]. Three major randomized studies shaped our current understanding of the potential benefits of CABG surgery in this scenario (Table 1): the “Veterans Administration (VA) Cooperative Study population” [10], the “European Coronary Surgery Study” (ECSS) [11], and the “Coronary Artery Surgery Study” (CASS) [12]. These trials included a small population by contemporary standards, and CABG was compared with the optimal medical treatment (OMT) at that time.

Overall, CABG surgery compared with OMT exerted a neutral effect on survivorship. Significantly limited by unpowered sample sizes, subanalyses suggested that surgery could be useful in reducing mortality only in selected groups: patients with more extensive coronary artery disease, depressed systolic function, or high-risk clinical features. For instance, CABG surgery reduced mortality in patients with left main disease in the 10-year (but not in the 18-year) follow-up of the VA population [10,16]. A similar tendency was detected in patients with multivessel disease plus left ventricular (LV) dysfunction included in the CASS [12] and those with multivessel disease (including proximal LAD disease) enrolled in the ECSS [11].

The effect of CABG surgery on CCS patient survival was further examined in a systematic review including seven trials with at least 10 years of follow-up [17]. Three quarters of recruited patients belonged to the three abovementioned trials that are summarized in Table 1. In this pooled analysis, CABG surgery was associated with higher survival rates in patients with extensive coronary artery disease (left main artery, three-vessel, or LAD disease) and those with left ventricular dysfunction. Regarding acute complications related to the surgical technique, this review obtained a perioperative MI rate of 7% and 3.2% of the patients assigned to the surgical group died within 30 days of surgery. These perioperative mortality rates were relatively variable in the three major randomized studies, from 1.4% in the CASS, 3.3% in the ECSS, and up to 5.8% in the VA population [12]. Information on perioperative stroke rates was not systematically available from the trials.

Leaving aside survival, CABG did not reduce MI (as a separate endpoint) in any of these three trials. CABG was initially superior to OMT alone for the purpose of improving quality of life indexes (such as angina relief, increased activity, and reduction in use of antianginal medications) but differences disappeared [10] or became much less apparent [15] 10 years after randomization.

In summary, results derived from trials carried out more than 30 years ago indicate that CABG surgery does not yield a dramatic long-term survival benefit compared with medical treatment in unselected patients with CCS. However, in specific populations with high-risk features such as those with extensive coronary disease or reduced left ventricular ejection fraction (LVEF), CABG surgery can provide enhanced survival compared with OMT alone. Again, these results must be interpreted in the context of the best available medical treatment during the years when the trials were performed.

## 3. Percutaneous Revascularization: “*The Unfulfilled Dream*”

Cardiac catheterization bolstered the development of interventional cardiology and marked a revolution in contemporary cardiology practice. This technique was first carried out by Werner Forssmann in 1929 [18], who self-cannulated his antecubital vein and advanced a urological catheter into his right atrium with fluoroscopic guidance. Thanks to this remarkable achievement, understanding of cardiac function progressed rapidly, heralding an exponential rise in the discovery of new invasive techniques.

In the late 1940s and early 1950s, non-selective techniques were developed to visualize the coronary arteries. These were indirect methods that consisted of filling the aortic root with high volumes of contrast to visualize the coronary arteries during diastole using conventional radiographs, yet only the proximal segments of the coronary arteries could be visualized. It was not until 1958 that Cleveland Clinic pediatric cardiologist Mason Sones performed the first selective coronary arteriogram, quite by accident [19] while attempting to inject contrast into the LV. The patient survived the procedure and visualization of the coronary arteries was far superior to that achieved by non-selective injection, so Sones proceeded to develop the selective coronary arteriography technique, which was clinically available in the mid-1960s [20]. By the late 1960s, Melvin Judkins created catheters that were specially shaped to reach the coronary arteries to perform selective coronary angiography [21].

In 1977, Andreas Grüntzig successfully performed the first percutaneous coronary intervention (PCI) as a transluminal balloon angioplasty [22], building on the work of Charles Dotter, considered the “father of interventional radiology,” on peripheral arteriosclerotic disease [23]. This achievement opened the door to a new era of interventional cardiology, obtaining a new therapeutic tool much less invasive than CABG surgery. However, the first trials comparing balloon angioplasty PCI vs. medical treatment in patients with CCS continued to show neutral and even negative results in terms of major events [24,25,26,27].

On top of the considerable incidence of acute coronary complications associated with balloon inflation, rates of restenosis after percutaneous transluminal coronary angioplasty (PTCA) were around 30% [28], and these factors were assumed to be the main obstacles to obtaining long-term benefits [29]. To overcome these hurdles, the first self-expanding metal stents were developed in the late 1990s [29]. However, despite optimization of anticoagulation and antiplatelet regimens, early thrombotic occlusion and in-stent restenosis remained a serious clinical problem, particularly in certain patient subgroups [30]. New biologic coatings capable of delivering drugs emerged quickly thereafter to counteract those issues. At the beginning of the 21st century, clinical trials of drug-eluting stents (DES) demonstrated a very low in-stent restenosis rate compared with bare-metal stents (BMS) [31,32], becoming the current mainstream therapy for coronary artery stenosis.

Advances in percutaneous revascularization procedures occurred in parallel with development of more efficient medical therapy based on optimization of antithrombotic therapy, strictest control of blood pressure, aggressive use of statins, and introduction of drugs aimed at modifying the natural history of the disease. Furthermore, standardization of radial artery as the preferred access for catheterization led to a significant decrease in the incidence of hemorrhagic events [33]. In this context, new randomized clinical trials were proposed to demonstrate the benefit derived from routine revascularization in patients with CCS. However, the results of these trials showed a lack of benefit from routine revascularization in addition to OMT in terms of survivorship.

Table 2 summarizes the four major trials comparing OMT plus revascularization (PCI or CABG) vs. OMT alone in CCS patient management: the “Clinical Outcomes Utilizing Revascularization and Aggressive Drug Evaluation” (COURAGE), the “Bypass Angioplasty Revascularization Investigation in Type 2 Diabetes” (BARI 2D), the “Fractional Flow Reserve-Guided Percutaneous Coronary Intervention versus Medical Therapy in Stable Coronary Disease” (FAME 2), and the “International Study of Comparative Health Effectiveness with Medical and Invasive Approaches” (ISCHEMIA).

The COURAGE trial [34] was the first comparative study carried out in this setting. A total of 2287 patients with angiographic evidence of IHD were randomized to OMT alone or on top of PCI. This trial failed to demonstrate significant differences in risk of death, MI, or other major adverse cardiovascular events (MACE) between the two groups. Regarding the COURAGE quality of life substudy [35], results somehow mimicked lessons derived from CABG trials. A greater proportion of the PCI group displayed significant improvements at 6 months after randomization, but these differences were no longer significant by 12 months and had vanished at 36 months.

In the BARI 2D trial [41], 2368 patients with type 2 diabetes mellitus and coronary artery disease (CAD) documented on angiography were randomized to undergo revascularization (PCI or CABG) on top of OMT or intensive OMT alone. Again, in this specific of patient subgroup with increased risk of complications during follow-up, no significant differences were observed in all-cause death and MACE rates [36].

In the FAME 2 trial [42], 1220 patients with at least one functionally significant stenosis as derived from the presence of a fractional flow reserve (FFR) ≤ 0.8 were randomly assigned to PCI plus OMT or OMT alone. The FFR-guided PCI strategy was more effective than OMT alone in reducing the risk of a combined endpoint (death, MI, or unplanned revascularization) but this benefit was restricted to the rate of unplanned revascularizations. This can be interpreted as a soft event, given that coronary anatomy was non-blinded for patients and physicians, which could have led to an overuse of revascularization procedures after any new episode of chest pain in patients with already known anatomically significant coronary disease. This observation persisted at 5-year follow-up, and again the effect on all-cause death was neutral [38].

There are no consistent data available from these trials about acute complications derived from PCI [43]. Only the COURAGE provided information on the rate of procedure-related infarction (3% in the PCI group vs. 0.7% in the OMT group) [34]. It could have been interesting to have more information to improve our understanding of the mechanisms behind the results obtained in terms of survival, although we must bear in mind that there are complications related to invasive procedures that do not cause immediate mortality but can shorten life expectancy (i.e., non-fatal acute complications, contrast-associated acute kidney injury, or bleeding).

The neutral results on major events derived from the three trials reviewed fueled the need to develop a strategy to better identify CCS patients who could benefit from coronary revascularization. In this regard, current guidelines recommend myocardial revascularization in CCS patients in two circumstances: (1) for symptomatic relief when a hemodynamically significant coronary stenosis is present and the patient remains symptomatic despite optimal medical therapy, and (2) to improve prognosis in the presence of anatomical or functional risk factors such as the presence of >10% ischemic myocardium by functional testing or FFR ≤ 0.8 [44]. These indications were drawn from two main observations, as detailed below.

Firstly, of the 2287 COURAGE patients, 314 were enrolled in the nuclear substudy [45]. Patients underwent single-photon emission computed tomography (SPECT) using exercise or vasodilator stress before treatment and during follow-up. It was shown that adding PCI to OMT resulted in significant ischemia reduction and symptom relief. Regardless of treatment assignment, the benefit was produced mainly in patients with large areas of ischemic myocardium (≥10%) in which the amount of ischemic myocardium was significant reduced.

Secondly, in 1005 patients with multivessel CAD included in the “Fractional Flow Reserve versus Angiography for Multivessel Evaluation” (FAME) trial [46,47], a physiology-based strategy (PCI only in lesions with FFR < 0.80) was superior to an anatomy-based strategy (PCI in lesions > 50% by angiography) at 1- and 2-year follow-up in terms of combined MACE. Differences were mainly driven by reductions in unplanned revascularizations. From 2 to 5 years, the risks for both groups developed similarly. No significant differences in all-cause mortality were observed during the entire follow-up period [48].

A recent meta-analysis based on available data from randomized controlled trials has emphasized that PCI revascularization in addition to OMT had no evident effect on all-cause mortality, cardiac mortality, or myocardial infarction [49]. Randomized trials have also been performed to compare the safety and efficacy of CABG vs. PCI in patients with CCS. Their principal finding was that survival was similar between both therapeutic groups, although CABG was superior to PCI in relieving angina and averting repeat revascularization procedures [50,51].

In the 1829 symptomatic patients with multivessel disease included in the “Bypass Angioplasty Revascularization Investigation” (BARI) trial, the overall 5-year survival rate was not significantly different between PCI and CABG, but survival was higher in the CABG group in diabetic patients [52].

As pointed out above, in the 2368 patients with type 2 diabetes included in the BARI-2D trial there was no significant difference in mortality and MACE rates between patients undergoing prompt revascularization (PCI or CABG) and those undergoing OMT. However, in patients in whom CABG was considered the optimal revascularization strategy (generally patients with extensive coronary disease), assignment to CABG was superior to OMT in terms of MACE rate reduction mainly by a lesser rate of MI. Nevertheless, in patients in whom PCI was recommended as the best revascularization strategy, assignment to PCI was not more effective than OMT. Based on these observations, current recommendations prioritize CABG over PCI or OMT alone in patients with diabetes and extensive atherosclerotic burden [44], especially if left main coronary artery disease [53,54].

**Figure 1:**
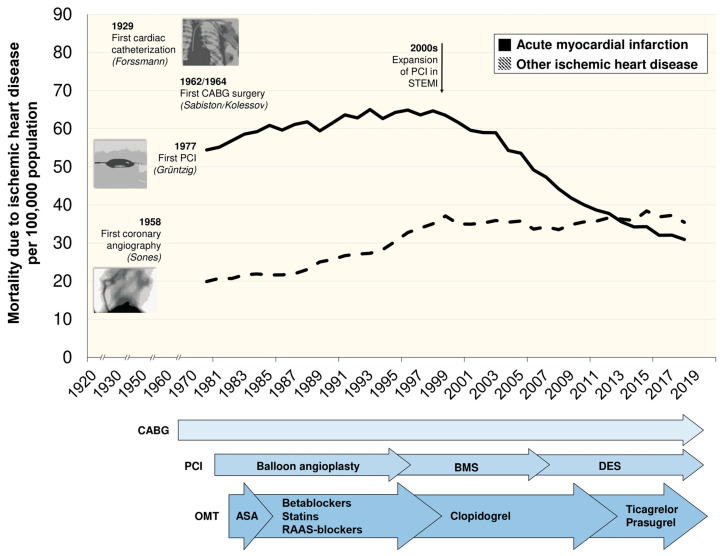
Time trends in mortality attributable to acute coronary syndrome (classified as acute myocardial infarction) and chronic coronary syndrome (classified as other ischemic heart disease) between 1980 and 2018, and main breakthroughs and research data in coronary revascularization and medical therapy during the last century. Data provided by the National Statistics Institute (I.N.E.) in Spain [56]. After the expansion of PCI in STEMI, a marked reduction in mortality was noted in acute myocardial infarction patients. This trend was not evident in patients with chronic coronary syndrome despite the widespread availability and use of CABG and PCI. Different groups of pharmacological therapies with beneficial effects on cardiovascular mortality have been incorporated into clinical practice during recent decades, and thus the “optimal medical treatment” has been improving over time. Abbreviations: ASA, Acetylsalicylic acid; BMS, Bare-metal stent; CABG, Coronary artery bypass grafting; DES, Drug-eluting stent; PCI, Percutaneous coronary intervention; RAAS, Renin–angiotensin–aldosterone system; STEMI, ST-segment elevation acute myocardial infarction.

In synthesis, by the 2010s indisputable evidence showing prolonged survival after PCI in acute coronary syndrome (ACS) [49] had still not been replicated in CCS patients (Figure 1) and the interpretation of results derived from trials carried out throughout the previous three decades was still questionable [55]. Beyond a certain superiority of CABG over PCI and OMT alone in patients with extensive coronary disease (especially those with diabetes) and of PCI over OMT alone in terms of soft events (unplanned procedures and symptom improvement), no significant reduction in mortality by revascularization in CCS patients was reported. Moreover, at that time published trials had only included patients undergoing catheterization and in whom the coronary anatomy was known. In routine practice, decisions generally have to be made on the basis of results obtained in non-invasive tests prior to catheterization.

Moreover, in parallel with the improvement in surgical and percutaneous revascularization techniques and technology, several pharmacological therapies for CCS treatment have been developed and implemented in the last few decades, such as acetylsalicylic acid and other antiplatelet agents (i.e., oral P2Y12 inhibitors), beta blockers, statins, and renin−angiotensin−aldosterone system blockers [1]. These five groups of drugs have been shown to reduce the risk of cardiovascular mortality, and their incorporation into routine practice over time is illustrated in Figure 1. Undeniably, the improvement in therapies that are considered “OMT” at each time makes comparison between historical trials challenging.

Altogether, this led to a consensus on the need for a definitive trial in which randomization to OMT alone or an invasive strategy on top of OMT should be driven by presence of ischemia in robust non-invasive stress imaging tests prior to catheterization [55]. In CCS patients with conclusive ischemia by non-invasive imaging, the derived results would definitively confirm or refute the benefit of revascularization in terms of hard events, namely death and MI. The long-awaited results of the ISCHEMIA trial have recently been published [39].

## 4. The Ischemia Trial: “*The Déjà-Vu Reality*”

The ISCHEMIA trial, one of the biggest publicly funded contemporary clinical research initiatives, was initiated in 2012 [57], and the results of this blockbuster clinical trial involving 5179 patients have recently been made available [39]. The main conclusion was that a routine invasive strategy in patients with CCS and evidence of moderate or severe ischemia on non-invasive testing did not confer any benefit in terms of reduction of MACE or death from any cause compared with OMT alone. In patients with angina at baseline, better quality of life and improved anginal relief was achieved with revascularization [40]. Nevertheless, the main objective of the trial, which was to prove clinical benefit in terms of hard outcomes, was not achieved. The neutral results of the ISCHEMIA trial have left the scientific community with conflicting interpretations, in what has been described as a “Rashomon effect” [58]. We will try to unravel some clues of this trial and address several points of uncertainty.

It is not unknown that the ISCHEMIA trial was initially designed to include only patients who underwent stress imaging techniques. The selected thresholds for stress nuclear imaging (≥10% ischemic myocardium), stress echocardiography (≥3/16 segments with stress-induced severe hypokinesis or akinesis), and stress cardiac magnetic resonance (CMR) (≥2 segments with stress-induced perfusion defects or ≥3 dobutamine-induced dysfunctional segments) were chosen and homogenized according to comparative definitions of moderate to severe ischemia based on patient prognosis [59]. However, due to patient recruitment difficulties, what authors described as “severe ischemia” on non-imaging exercise electrocardiogram (ECG) testing was accepted in 2014 as another criterion for patient inclusion in the trial [57,60]. Even though exercise ECG testing inclusion criteria were indeed more restrictive than in real-life clinical practice, the inclusion of this non-imaging, “*not so reliable*” stress technique may have altered the results of the trial [61]. While wall motion abnormalities and stress-induced perfusion defects are usually due to ischemia, in some cases no significant epicardial stenoses are noted on coronary angiogram. Nevertheless, the overall performance of non-invasive imaging tests for either ruling in or ruling out significant CAD is superior to the performance of the exercise ECG testing [1].

The crossover rate in the ISCHEMIA trial [39] has also been discussed as a potential confounding factor: 21.6% of patients assigned to invasive strategy did not undergo revascularization, and 21% of patients assigned to conservative strategy finally underwent revascularization. Since the analysis was by intention to treat, these crossovers could have interfered with the results.

**Figure 2:**
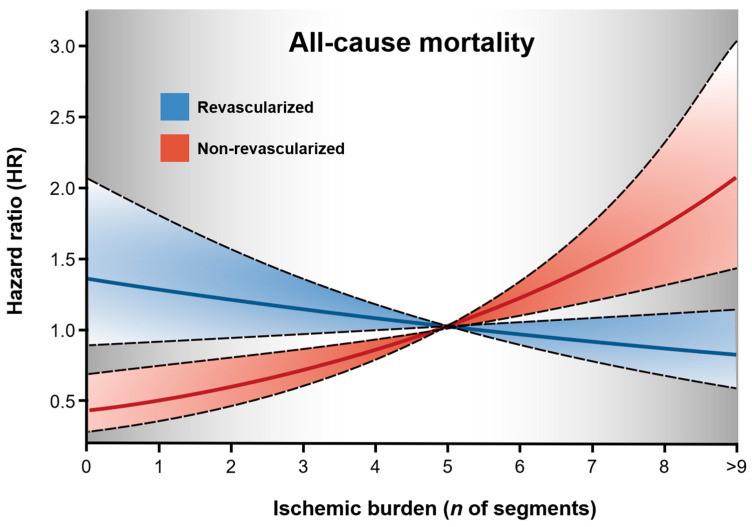
Effect of stress CMR-related revascularization on all-cause mortality in a large real-life registry of patients with known or suspected CCS. Adapted from Marcos-Garces et al. [62] following the STM Permissions Guidelines (2014). Results derived from a large registry of patients with known or suspected CCS undergoing stress CMR. In non-revascularized patients, the risk of all-cause mortality increased in parallel with ischemic burden (number of segments with inducible perfusion defects on stress first-pass perfusion) but the opposite trend was observed in revascularized patients, with lines crossing at five ischemic segments. Shaded areas correspond to patient profiles that were “probably” not included in the ISCHEMIA trial. Abbreviations: CCS, Chronic coronary syndrome; CMR, Cardiac magnetic resonance.

Another issue worth raising is the relatively low event rate reported during the trial. Thresholds for imaging techniques were adjusted to reach a rate of cardiac death or non-fatal myocardial infarction of 5% per year [57]. However, although the primary endpoint was downgraded to also include hospitalization for unstable angina, heart failure, or resuscitated cardiac arrest, the final event rate was roughly 4% per year [39]. This difference compared with a real-life setting may reflect the reluctance of clinicians to randomize patients with the most severe ischemia, i.e., those at the highest risk. Additionally, following predefined criteria, patients with suspected left main stem disease by computed tomography (CT) were also excluded from the analysis. Overall, it could be argued that the vast majority of the study group included in the trial was made up of patients without “really severe” ischemia; a relatively low-intermediate risk scenario in which large improvements in terms of hard events cannot be expected. Thus, CCS patients at highest risk (those who could benefit most from revascularization) did not show up in the overview of the ISCHEMIA trial (Figure 2). Taken out of context, the initial take-home message “*medical management for all*” could lead to future over-skepticism among decision-makers and underuse of invasive resources in these high-risk subsets.

On the other hand, as predefined in the ISCHEMIA trial, patients with known or suspected CCS but without evidence of ischemia or with mild ischemia in stress tests were not supposed to appear on the other side of the picture (Figure 2). The consensus now is to be very restrictive in use of invasive procedures in this subset. However, patients in whom the extent of ischemia does not fulfill criteria of severity or remains unknown represent a high proportion of CCS patients submitted to revascularization during the last few decades (and probably nowadays) [45]. Conclusions on the effect (beneficial, neutral, or deleterious) of revascularization in this type of CCS patients cannot be derived from the trial.

In summary, the ISCHEMIA trial has been the last of a *déjà vu* of studies trying (and failing) to confirm a benefit on hard endpoints with revascularization in CCS, in this case after purported demonstration of at least moderate ischemia on non-invasive testing. Whether stricter inclusion criteria (e.g., higher thresholds for ischemia) and the adherence to only robust stress imaging techniques could have modified the results is mere speculation, but effort should be made to further explore and understand this possibility. Despite all the limitations imposed by a lack of randomization, information derived from large registries carried out during the last two decades can help gain the full picture, in which all characters are present. Sometimes this can portend an awareness of uncomfortable truths.

## 5. Real-Life Registries: “*The Uncomfortable Truth*”

Randomized trials are one of the cornerstones of progress in medicine, but their inherent limitations should also be kept in mind [63]. In this sense, care should be taken that the lack of benefit in terms of reducing hard events by revascularization in CCS patients reported in trials does not trigger an excessive increase in conservative management. Undoubtedly, an unknown proportion of very high-risk patients are excluded from trials a priori (not randomized due to caution of investigators, i.e., selection bias). Others, due to the prespecified design [63], are excluded a posteriori (upon detection of severe anatomical disease). Moreover, on the other side of the picture, it seems clear that over recent decades (and probably even now) a certain overuse of invasive therapies may have occurred in the setting of CCS [64]. The implications of this last impression on outcomes cannot be interpreted from the results of randomized trials because these patients were not enrolled. These observations suggest the need for careful interpretation of the information provided, not only by randomized trials but also by registries [65,66,67,68,69,70] to guide appropriate decision-making.

Registries using stress echocardiography to assess inducible wall motion abnormalities with dobutamine [65,66] or myocardial perfusion defects with dipyridamole [67] suggested an improved prognosis associated with revascularization in patients with significant myocardial ischemia.

Stress nuclear imaging by either SPECT or positron emission tomography (PET) has played a crucial role in diagnosis and prognostic assessment of CCS patients. Hachamovitch and colleagues conducted one of the most influential studies in this field. In a single-center observational cohort of 10,627 patients with suspected CCS who underwent stress myocardial perfusion SPECT [68], and afterwards in a subsequent update of the same cohort with a total of 13,969 patients [69], revascularization was associated with prolonged survival in cases of moderate to severe ischemia. Current guidelines have embraced the 10% threshold of ischemic myocardium established by the authors [44]. On the other hand, the use of revascularization in patients below the 10% threshold was related to a higher risk of all-cause death. A link with extent of scarring was also suggested, somewhat supportive of a role for residual myocardial viability in the use of invasive resources. In a more recent and contemporary registry including 16,029 patients undergoing perfusion PET, survival benefit with revascularization was observed in patients with evidence of myocardial ischemia [70].

Stress CMR permits comprehensive diagnostic and prognostic evaluation of patients with known or suspected CCS. This is now the gold standard for accurate quantification of wall motion abnormalities, LVEF, and necrosis (using late gadolinium enhancement sequences). Moreover, the presence, location, and extent of myocardial ischemia can be reliably determined by assessing inducible wall motion abnormalities, or more frequently perfusion defects by stress perfusion CMR. The prognostic value of each of these parameters has been extensively demonstrated [71,72,73,74,75,76,77].

Over the last couple of decades, our group has compiled a large prospective registry that has sequentially demonstrated the value of stress CMR for diagnosis, risk stratification, and decision-making in patients with known or suspected CCS [62,73,74,75,77,78]. In the most recent update [62], we analyzed 6389 consecutive CCS patients undergoing stress perfusion CMR and focused on the most unarguable event, namely all-cause death. The effect of CMR-related revascularization on all-cause mortality was explored. During a 5.75-year median follow-up, 717 (11%) deaths were documented. More extensive ischemic burden was independently related to all-cause mortality (4% increased risk of all-cause death per 1-segment increase in ischemic burden). In a strictly 1:1 matched population, revascularization was associated with less all-cause mortality in patients with very extensive CMR-related ischemia (>5 segments), whereas in those without or with non-extensive ischemia (5 or less segments) revascularization seemed to exert a neutral or even deleterious effect. Similar tendencies have been reported by other authors [79].

Based on our results, Figure 2 illustrates how patient profiles in which revascularization could influence most (positively in those with extensive ischemia and negatively in those without or with only mild ischemia) have generally been excluded from trials. Unsurprisingly, the effect in randomized patients (those in between) has been repeatedly found to be neutral in terms of hard events.

Ultimately, the results obtained by our group [62] and by similar registries [68,69] indicate that gains in terms of survivorship from revascularization cannot be expected (and the effect could even be deleterious) in patients without “really severe” ischemia. Our findings parallel recent research reporting an increased all-cause mortality risk in patients undergoing coronary angiography, despite the absence of significant coronary stenosis (>50%) by coronary computed tomographic angiography (CT) [80]. Similarly, the “Swedish Coronary Angiography and Angioplasty Registry” (SCAAR) analyzed 23,860 patients undergoing PCI for stable angina; in only 3367 (14%) of them was PCI guided by FFR, but compared with unselective revascularization this strategy was associated with a lower risk of long-term mortality [81].

Thus, anatomically and physiologically based results seem to suggest that as a rule, invasive management should be reserved for patients at the highest risk because overuse of these resources could potentially exert deleterious effects on low-risk CCS patients. In this setting, risky and technically challenging interventions should probably be avoided, and use of revascularization has to be justified by other motivations such as symptom relief or an attempt to reduce the risk of softer events; moreover, it should be remembered that the evidence is controversial even for this purpose. Whereas CABG and PCI trials suggested a significant (though transient) anginal relief associated with revascularization, the “Objective Randomised Blinded Investigation with Optimal Medical Therapy of Angioplasty in Stable Angina” (ORBITA) trial [82] failed to demonstrate a sustained increase in exercise time in patients with a physiologically significant lesion when PCI was compared with OMT alone.

Whereas invasive therapies may have been overused in the setting of CCS in the past [64], caution should be taken to ensure that the lack of benefit in reducing hard events with revascularization observed in randomized trials does not tip the balance towards overly conservative management. Results of large real-life registries might be useful as a reminder that invasive management might be reasonable when severe ischemia is detected in non-invasive tests in the work-up of CCS; indeed, this strategy can even lead to prolonged survival. When attempting to justify use of revascularization aimed at prolonging survival, the threshold applied should be flexible and interpreted in tandem with clinical presentation. Fixed interpretations should not be established, because optimal cut-off points depend on both the type of stress and cardiac imaging used. Summarizing, data derived from large registries using different stress imaging modalities strongly suggest that to achieve a significant reduction in mortality risk, extensive ischemic burden must be demonstrated by one of the available non-invasive cardiac imaging techniques.

However, the potential therapeutic implications of registries in the field of CCS should be interpreted with great caution and we have to be aware of the limitations of this type of approach. The largest registries commented on above [62,72] refer to unselected populations with known or suspected CCS submitted to undergo stress imaging and thus do not substantiate that a strategy based on stress imaging-guided revascularization is superior to another where this technique is not applied. Even though strict propensity matching methods can be used in registries to match revascularized to non-revascularized patients, this statistical exercise hardly contemplates the myriad of factors relevant in the impact of revascularization on the outcomes of CCS patients that could only be correctly addressed by a true randomized study.

## 6. And Now What?

**Figure 3:**
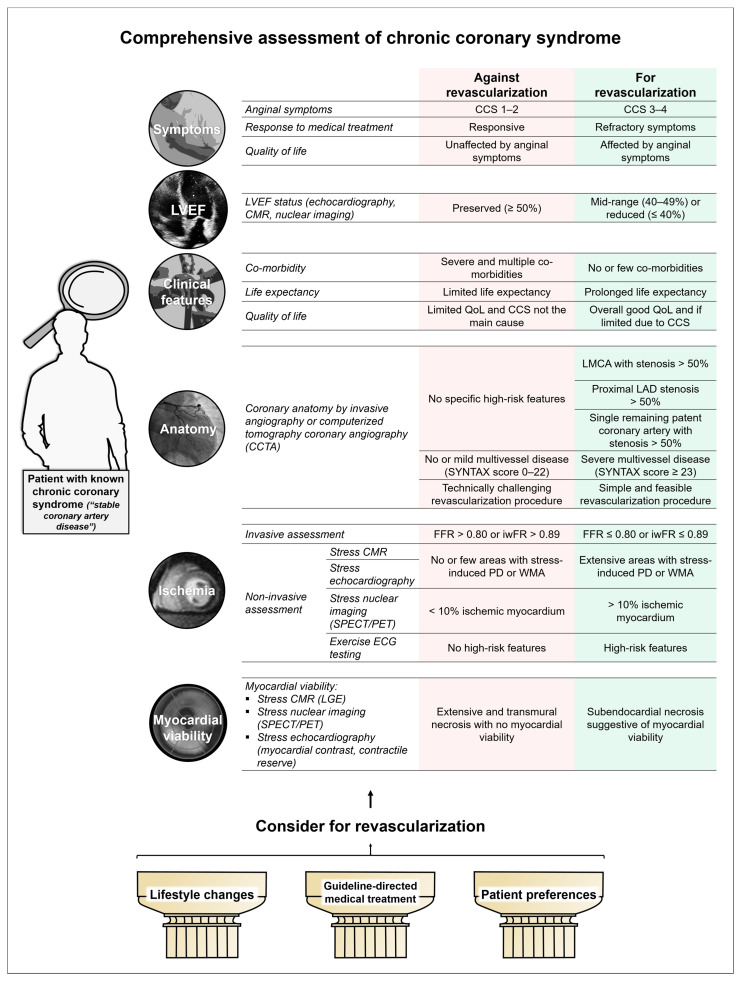
Comprehensive management of chronic coronary syndrome. Lifestyle changes (if needed) and guideline-directed medical treatment must be implemented; patient preferences have to be carefully considered. A comprehensive assessment of symptoms, clinical characteristics of patients, LVEF, and of the anatomical and/or physiological repercussion of coronary lesions will be needed to appropriately guide the use of revascularization in CCS. Abbreviations: CCS, Canadian Cardiovascular Society angina score; CMR, Cardiac magnetic resonance; FFR, Fractional flow reserve; LAD, Left anterior descending artery; LGE, Late gadolinium enhancement; LMCA, Left main coronary artery; LVEF, Left ventricular ejection fraction; QoL, Quality of life; SPECT, Single-photon emission computed tomography; PD, Perfusion defect; PET, Positron emission tomography.

Drawing from the results of randomized clinical trials and observational data, recent myocardial revascularization [44] and CCS management [1] guidelines provide a fairly up-to-date framework on why, when, and whom to revascularize in the CCS setting. Decision-making is unlikely to become generalized to all patients, or even to be a straightforward process considering only a limited number of factors. The intrinsic complexity of CCS itself and the broad pathophysiological and clinical spectrum necessitates comprehensive assessment of CCS patients (Figure 3).

### 6.1. Initial Assessment of CCS

By far the most valuable information for establishing a correct diagnosis of CCS derives from clinical history, in which correct interpretation of patients’ symptoms is key. Once suspected, the definitive diagnosis of CCS can be confirmed by either functional (stress echocardiography, stress nuclear imaging, stress CMR, or exercise ECG testing) or anatomical (invasive angiography or coronary computed tomography angiography (CCTA)) testing [1]. Beyond diagnosis, these tools also provide relevant prognostic information and exert a decisive influence on decision-making (i.e., the use of revascularization).

A strategy based on non-invasive testing is effective and reduces costs [83]. Indeed, the initial use of stress CMR correlates with fewer referrals for invasive coronary angiography with no impact on patient outcomes [79]. Nagel et al. recently demonstrated in CCS patients that a strategy based on the use of stress CMR was associated with a lower incidence of coronary revascularization than FFR and was non-inferior to FFR with respect to MACE occurrence [84].

Some patients with very high pretest probability are referred for an invasive coronary angiography without further previous assessment. Furthermore, based on the futility of revascularization in CCS patients included in trials, some authors have suggested an entirely symptom-guided or anatomy-guided management [44,85], obviating the use of stress imaging. In this case, non-invasive functional data will not be available at the time of interpreting the repercussion of the detected coronary lesions. Avoiding hasty decisions at this point is crucial, and if the potential benefit of revascularization is unclear, the use of invasive functional testing such as FFR or an instantaneous wave-free ratio (iFR) can be extremely helpful. Other angiographically derived parameters such as quantitative flow ratio (QFR) correlate well with FFR and iFR and can help discriminate functionally significant stenosis [86]. However, the invasiveness and potential complications of this approach should be kept in mind when considering initial non-invasive vs. initial invasive testing.

### 6.2. Left Ventricular Ejection Fraction

Apart from coronary anatomical or functional data, assessment of systolic function (and of LVEF as its most universally used proxy) constitutes a mandatory step in the work-up of CCS. The deleterious prognostic repercussion of depressed LVEF, and its utility as a parameter for guiding decisions are globally recognized not only in the field of CCS but also in all cardiovascular diseases. Based on the results of pivotal trials comparing surgical revascularization vs. OMT several decades ago, an almost unanimous belief has persisted that in patients with severely reduced LVEF complete revascularization should always be sought, preferably with CABG [87]. Although easily understandable and biologically plausible, the role of myocardial viability to guide the use of revascularization in patients with reduced LVEF is still unclear [88]. In spite of the greater improvement in LVEF expected in patients with myocardial viability, no differences in long-term survival have been demonstrated [89]. However, in patients with depressed LVEF, assessment of residual myocardial viability as well as the extent and location of ischemia contribute key information for decision-making. The current recommendation is to completely revascularize patients with severely reduced LVEF if technically feasible.

### 6.3. The Need for OMT

Every emphasis should be placed on the need for OMT in all patients with CCS [90]. This treatment can be categorized into two subsets of measures.

First, certain therapies such as statins [91], antihypertensive, antidiabetic, and antiplatelet drugs can be helpful to control cardiovascular risk factors, slow or even reverse the progression of atheromatous disease, and prevent blood clot formation. Prolonged dual antiplatelet therapy has been shown beneficial in a subset of patients with high ischemic risk [92]. Lifestyle changes and cardiac rehabilitation could also be considered as part of integrative medical management and are generally recommended [1]. Secondary prevention targets should be sought and achieved whenever possible to improve prognosis. This objective has proven elusive so far despite the efforts of scientific societies in recent years [93].

Secondly, additional pharmacological treatments such as beta blockers can be implemented to control anginal symptoms, some of them even conferring a prognostic benefit on top of symptomatic relief [94]. A wide range of drugs are available, and other alternatives such as the coronary sinus reducer device [95] are emerging for management of refractory angina.

If patients remain symptomatic after comprehensive OMT implementation, revascularization may be indicated despite absence of high-risk features. Clinicians should probe into the severity of patients’ symptoms, for which purpose the Canadian Cardiovascular Society (CCS) angina score is widely applied. Despite the recent challenge to this paradigm in the ORBITA trial [82], the main body of evidence supports this management [96].

Elderly, fragile, and co-morbid patients have traditionally been excluded from trials evaluating the effect of revascularization in CCS. Given the unknown benefit in terms of prognosis in these populations, use of aggressive strategies needs to be individualized, requiring in-depth knowledge of patients and their circumstances. In any case, age cannot be the only factor to be considered, and undoubtedly OMT must always be applied and prioritized [97].

### 6.4. Revascularization and Patient Profiles

Taken together, all the data gathered on anginal symptoms, LVEF, functional assessment, anatomical characteristics, and patient expectations and preferences should enable us to recommend for or against revascularization.

**Figure 4:**
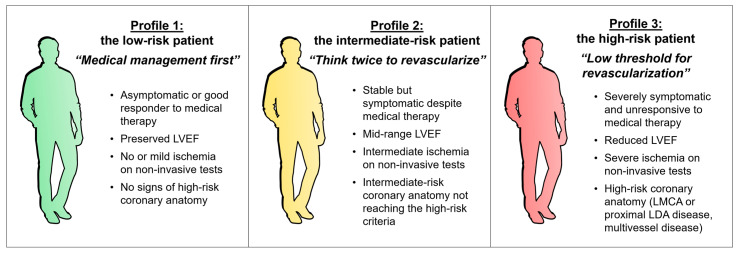
Patient profiles in chronic coronary syndrome to guide a revascularization strategy. Based on symptoms, LVEF, and the anatomical and/or physiological repercussion of coronary lesions, three patient profiles can be established. In patient profiles 1 (“low-risk”, medical management first) and 3 (“high-risk”, low threshold for revascularization), decision-making is relatively easy. In patient profile 2 (“intermediate-risk”, think twice to revascularize), factors depicted in Figure 3 have to be cautiously considered. Abbreviations: CMR, Cardiac magnetic resonance; LAD, Left anterior descending artery; LGE, Late gadolinium enhancement; LMCA, Left main coronary artery; LVEF, Left ventricular ejection fraction; PD, Perfusion defect.

Several patient profiles may be envisioned, as depicted in Figure 4.

The first profile is the low-risk patient, or “medical management first”. This group integrates asymptomatic cases or good responders to medical therapy in which no high-risk features are present. OMT should be prioritized, and the use of revascularization has to be the exception.

In the third profile, the high-risk patient with uncontrolled symptoms or high-risk features such as extensive coronary disease (left main stem, proximal LAD, or multivessel disease), depressed LVEF (<50%), or severe ischemia on non-invasive testing (stress-induced wall motion abnormalities or large perfusion deficit), a “low threshold for revascularization” should be applied and if reasonably feasible it has to be recommended.

However, a substantial number of cases lie somewhere in between these two well-defined poles and represent intermediate-risk patients, typified by mixed features such as persisting but non-limiting symptoms or intermediate anatomical or functional risk features. These patients are probably best represented in the ISCHEMIA trial, and we should therefore “think twice to revascularize”, even though it can be a reasonable option after weighing risks and benefits. It is mainly in this scenario where a thorough evaluation of clinical, functional, and anatomical features represented in Figure 3 can help most to make the appropriate decision. Nevertheless, a robotic answer to the question “should I revascularize my patient?” is unrealistic, and honest communication with our patients, deep knowledge of the disease, and further research are of the utmost importance.

## 7. Conclusions

In recent decades, a *déjà vu* of studies have tried unsuccessfully to demonstrate a benefit of revascularization to survivorship in CCS. Our dreams (of benefit to hard endpoints) have to remain unfulfilled for now. The experience of all these years and data derived from large real-life registries reveal, on the one hand, the uncomfortable truth of the potentially harmful effects of the overuse of revascularization in low-risk populations. Yet on the other hand, the initial “*medical management for all*” message that could arise from this may not apply to a substantial number of high-risk patients (generally not included in trials) for whom appropriate use of revascularization could exert considerable benefits, even in life expectancy. In conclusion, our hopes remain intact but we must continue to accept ambiguity, correctly interpret trials and registries, embrace decisions tailored to the individual, and continue to put patients first.

## Figures and Tables

**Table 1:** All-cause mortality in the main trials comparing medical treatment plus coronary artery bypass grafting (CABG) surgery vs. medical treatment alone in patients with chronic coronary syndrome.

Trials	No. of Patients			Follow-Up (Years) *	Annualized All-Cause Mortality Rate (%/Year)		
	CABG	OMT	Total		CABG	OMT	*p*
VA [10]	332	354	686	18	3.9%	3.7%	0.6
ECSS [11]	394	373	767	12	1.9%	2.4%	0.04
CASS [12,15]	390	390	780	10	1.8%	2.1%	0.25

* Median follow-up in years. Abbreviations: CABG, Coronary artery bypass grafting; CASS, Coronary Artery Surgery Study; ECSS, European Coronary Surgery Study; OMT, Optimal medical treatment; VA, Veterans Administration Cooperative Study population.

**Table 2:** All-cause mortality in the main trials comparing medical treatment plus revascularization (percutaneous coronary intervention (PCI) or CABG) vs. medical treatment alone in patients with chronic coronary syndrome.

Trials	No. of Patients			Follow-Up (Years) *	Annualized All-Cause Mortality Rate (%/Year)		
	Rev	OMT	Total		Rev	OMT	*p*
COURAGE [34,35]	1149	1138	2287	4.6	1.6%	1.8%	0.38
BARI 2D [36,37]	1176	1192	2368	5.3	2.2%	2.3%	0.97
FAME 2 [38]	447	441	888	5	1%	1%	NS
ISCHEMIA [39,40]	2588	2591	5179	3.2	2.8%	2.6%	0.67

* Median follow-up in years. Abbreviations: BARI 2D, Bypass Angioplasty Revascularization Investigation in Type 2 Diabetes; COURAGE, Clinical Outcomes Utilizing Revascularization and Aggressive Drug Evaluation; FAME 2, Fractional Flow Reserve-Guided Percutaneous Coronary Intervention versus Medical Therapy in Stable Coronary Disease; ISCHEMIA, International Study of Comparative Health Effectiveness with Medical and Invasive Approaches; NS, Non-significant; OMT, Optimal medical treatment; Rev, Revascularization.
